# Supplementary material for: Venomix: a simple bioinformatic pipeline for identifying and characterizing toxin gene candidates from transcriptomic data
Source: PeerJ. 2018 Jul 31;6:e5361. doi: 10.7717/peerj.5361 (PMC6074769; doi:10.7717/peerj.5361)
Supplement: Supplemental Information 6 [file peerj-06-5361-s006.gz › FinalOutput_1E-6/Plancitoxin-1_1/finaltree.pdf]

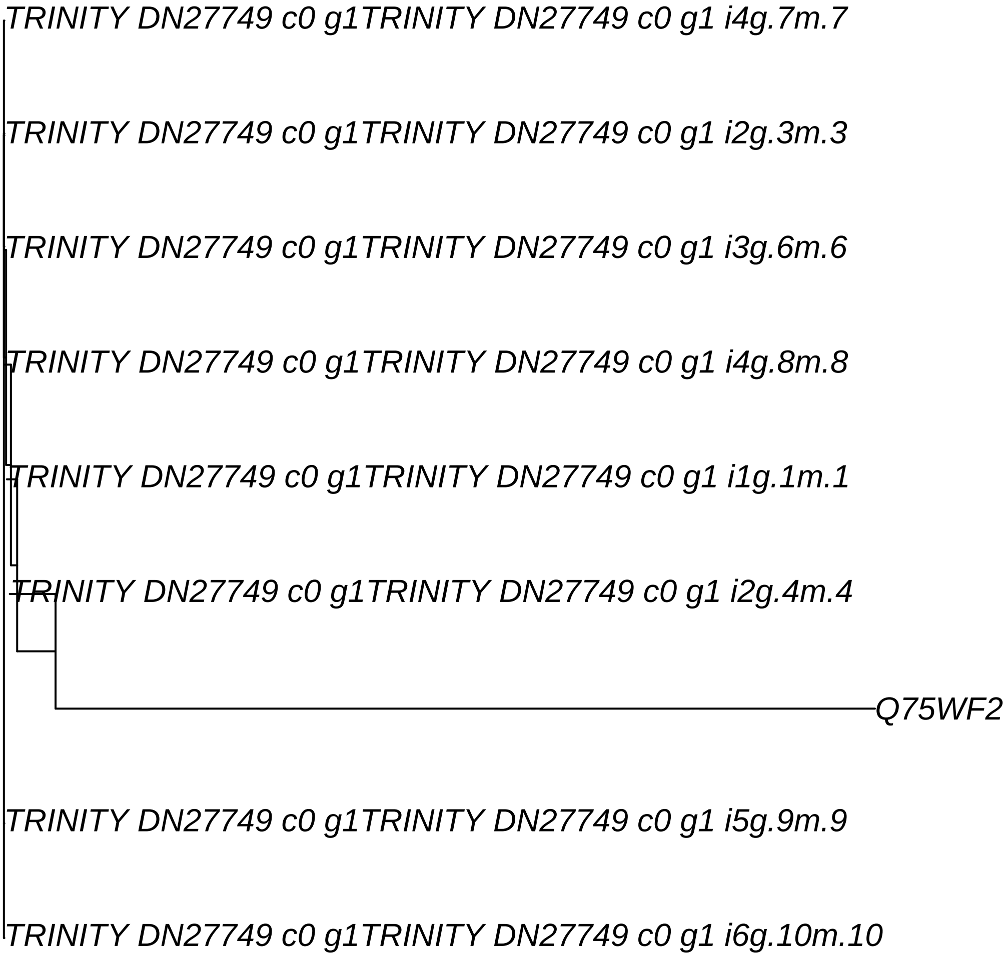
TRINITY DN27749 c0 g1TRINITY DN27749 c0 g1 i4g.7m.7
TRINITY DN27749 c0 g1TRINITY DN27749 c0 g1 i2g.3m.3
TRINITY DN27749 c0 g1TRINITY DN27749 c0 g1 i3g.6m.6
TRINITY DN27749 c0 g1TRINITY DN27749 c0 g1 i4g.8m.8
TRINITY DN27749 c0 g1TRINITY DN27749 c0 g1 i1g.1m.1
TRINITY DN27749 c0 g1TRINITY DN27749 c0 g1 i2g.4m.4
Q75WF2
TRINITY DN27749 c0 g1TRINITY DN27749 c0 g1 i5g.9m.9
TRINITY DN27749 c0 g1TRINITY DN27749 c0 g1 i6g.10m.10
